# Bedside Diaphragm and Lung Ultrasound for Predicting Liberation from Mechanical Ventilation: A Systematic Review with Clinical Synthesis

**DOI:** 10.3390/jcm15103877

**Published:** 2026-05-18

**Authors:** Amro Al Radaideh, Ghaith Batarseh, Ibrahim Alfarrajin, John Paul Fox, Mu’nis Al-Radaideh, Joseph Joji, Nirav Mistry

**Affiliations:** 1 Saint Michael’s Medical Center, Newark, NJ 07102, USA; gbatarseh@primehealthcare.com (G.B.); jfox1@primehealthcare.com (J.P.F.); jjoseph3@primehealthcare.com (J.J.); nmistry@primehealthcare.com (N.M.); 2 Ascension Saint Agnes Hospital, Baltimore, MD 21229, USA; ibrahim.alfarrajin@ascension.org; 3 Faculty of Medicine, Jordan University of Science and Technology, Irbid 22110, Jordan; msalradaideh20@med.just.edu.jo

**Keywords:** diaphragm ultrasound, lung ultrasound, extubation failure, weaning, mechanical ventilation, spontaneous breathing trial

## Abstract

**Background:** Failure of liberation from mechanical ventilation remains common despite successful spontaneous breathing trials (SBTs) and is associated with increased morbidity, prolonged ICU stay, and mortality. We aimed to evaluate the role of diaphragm and lung ultrasound for predicting composite liberation failure, including SBT failure and post-extubation failure, in adults. **Methods:** We conducted a systematic review with descriptive synthesis in accordance with PRISMA-DTA guidelines. The review protocol was developed a priori but was not prospectively registered. MEDLINE, EMBASE, and Cochrane CENTRAL were searched from inception to 20 January 2026. Adult studies evaluating diaphragm ultrasound (diaphragm thickening fraction [DTF], diaphragmatic excursion [DE]) and/or lung ultrasound in patients undergoing SBTs were included. Risk of bias was assessed using Quality Assessment of Diagnostic Accuracy Studies-2 (QUADAS-2). **Results:** Six studies (*n* = 430) were included. DTF demonstrated the most consistent association with liberation failure (AUC range 0.64–0.99; sensitivity 78–100%). DE showed a similar but less consistent association (AUC range 0.77–0.86). Elevated lung ultrasound scores—reflecting aeration loss from cardiogenic edema, acute respiratory distress syndrome (ARDS), atelectasis, or pneumonia—were associated with extubation failure. **Conclusions:** DTF shows potential clinical utility as an adjunct for predicting liberation outcomes. LUS offers complementary insight into aeration loss regardless of etiology. Standardization of measurement protocols and larger prospective studies are needed.

## 1. Introduction

Liberation from invasive mechanical ventilation represents a critical milestone in intensive care management, yet extubation failure remains common and is consistently associated with increased morbidity, prolonged intensive care unit (ICU) and hospital length of stay, and higher mortality [1]. Reported rates of extubation failure range from 10% to 20% in general ICU populations and may be even higher in high-risk subgroups such as patients with chronic respiratory disease, cardiac dysfunction, or prolonged mechanical ventilation. Importantly, both premature extubation and unnecessary delays in liberation carry risks, emphasizing the need for accurate and physiologically meaningful predictors of readiness for extubation.

Conventional weaning parameters, including SBTs and indices such as the rapid shallow breathing index (RSBI) and negative inspiratory force (NIF), remain the foundation of clinical liberation assessment and are widely validated in diverse ICU populations [1]. These indices are objective, readily obtainable at the bedside, and continue to inform extubation decisions in routine practice. However, liberation failure is multifactorial and may be influenced by respiratory muscle dysfunction, impaired lung aeration, cardiac dysfunction, neurological impairment, renal dysfunction, and other systemic factors. No single parameter, including RSBI or NIF, fully captures this complexity, and prediction of extubation outcomes remains imperfect.

Bedside ultrasound has been proposed as a potentially complementary, non-invasive tool that may provide additional physiologic information during liberation assessment. Diaphragm ultrasound—particularly DTF and DE—may offer insight into respiratory muscle function. Lung ultrasound (LUS) may provide information regarding lung aeration. The prior systematic review and meta-analysis by Llamas-Álvarez et al. (2017) reported a summary AUC of 0.87 for DTF across 19 studies [1]; however, that review was limited by a search cutoff of November 2016, heterogeneous outcome definitions that included studies without post-extubation follow-up, and the absence of a formal risk of bias assessment using QUADAS-2. The present review extends the prior work in four specific ways: (1) it incorporates five studies published after the 2016 search cutoff, including two large prospective cohorts from 2024; (2) it restricts eligibility to composite liberation failure outcomes—encompassing both SBT failure and post-extubation failure—providing greater clinical homogeneity; (3) it applies a full QUADAS-2 risk of bias assessment with a traffic-light summary; and (4) it presents, for the first time in a systematic review format, a structured diagnostic accuracy table with AUC, sensitivity, specificity, PPV, and NPV per study per parameter, enabling direct cross-study comparison. The current evidence base remains preliminary and the findings should be interpreted accordingly.

## 2. Materials and Methods

### 2.1. Search Strategy

This review followed PRISMA-DTA reporting guidelines, and the completed PRISMA checklist is provided in the Supplementary Material. MEDLINE, EMBASE, and Cochrane CENTRAL were searched from inception to 20 January 2026 using combinations of keywords and Boolean operators including “diaphragm ultrasound,” “lung ultrasound,” “extubation,” “weaning,” and “mechanical ventilation.” The full search strategy is provided in the Supplementary Materials.

### 2.2. Study Selection

We included adult studies evaluating diaphragm ultrasound (DTF, DE) and/or lung ultrasound in patients undergoing SBTs with extractable diagnostic accuracy data. Exclusion criteria included pediatric populations, absence of relevant outcomes, lack of ultrasound assessment, and inability to extract a 2 × 2 table for sensitivity and specificity. Two reviewers independently screened studies; disagreements were resolved by consensus. For consistency throughout this review, the following terminology is used: “liberation failure” refers to any failure of the process of discontinuing invasive mechanical ventilation, encompassing both SBT failure and post-extubation failure; “SBT failure” refers specifically to failure during the spontaneous breathing trial prior to extubation; and “extubation failure” refers to post-extubation respiratory distress requiring reintubation or escalation to non-invasive ventilation within 48 h. Where included studies used “weaning failure” as their primary endpoint, the specific definition used by each study is noted in Table 1.

This review includes a different study selection from Llamas-Álvarez et al. (2017) [1] because of our more recent search date (to January 2026), our restriction to composite liberation failure outcomes, and our exclusion of studies enrolling only post-SBT-success patients, which introduces spectrum bias.

### 2.3. Data Extraction and Analysis

Extracted variables included study design, population, ICU type, SBT method, ultrasound modality and timing, diagnostic thresholds, and outcome definitions. Diagnostic accuracy metrics (sensitivity, specificity, AUC, PPV, NPV) were extracted or derived from reported data.

DTF measurement methodology: A high-frequency linear probe (7–15 MHz) is placed at the zone of apposition of the right hemidiaphragm (8th–10th intercostal space, midaxillary line). Diaphragm thickness is measured at end-expiration (DT-exp) and end-inspiration (DT-insp). DTF = (DT-insp − DT-exp)/DT-exp × 100%, averaged over three consecutive breaths.

Results were synthesized descriptively rather than through pooled meta-analysis because of substantial heterogeneity in ultrasound protocols, diagnostic thresholds, SBT techniques, and outcome definitions. Risk of bias was assessed using QUADAS-2 across four domains: patient selection, index test, reference standard, and flow and timing.

## 3. Results

### 3.1. Study Selection

**Figure 1:**
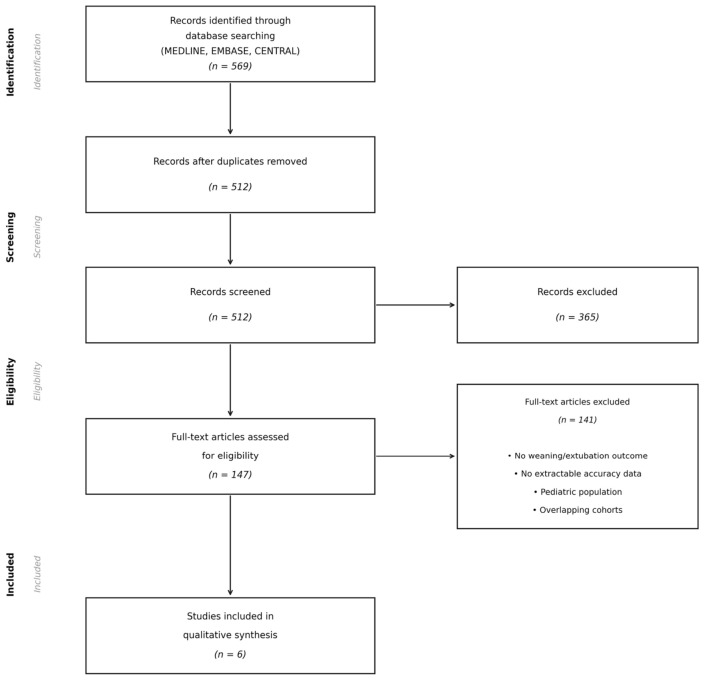
PRISMA flow diagram of study selection. Records identified through database searching (MEDLINE, EMBASE, CENTRAL). Duplicates removed, titles/abstracts screened, full-text assessed for eligibility, and 6 studies included in qualitative synthesis.

The study selection process is illustrated in Figure 1. The search identified 569 records; 147 underwent full-text review; six were included in the final qualitative synthesis.

### 3.2. Study Characteristics

The included studies were published between 2017 and 2024 and comprised 430 adult patients (Table 1). Most were prospective observational cohorts in mixed medical-surgical ICUs; one was conducted in a cardiothoracic ICU (Genty et al. 2022) [5]. Ultrasound assessments were performed before, during, or after SBTs, reflecting variability in clinical practice.

### 3.3. Risk of Bias Assessment

**Figure 2:**
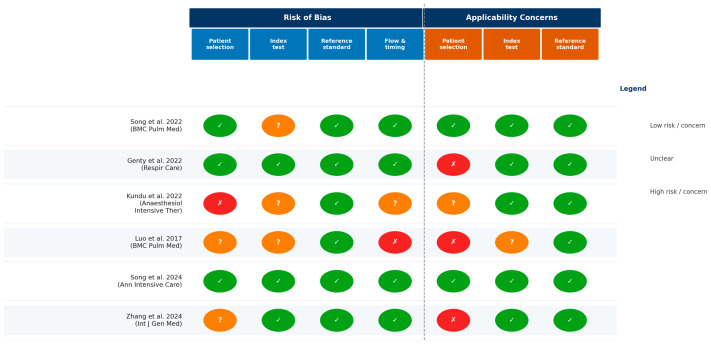
QUADAS-2 risk of bias and applicability concerns for all six included studies [2,3,4,5,6,7]. Green (✓) = low risk/concern; amber (?) = unclear; red (×) = high risk/concern.

Risk of bias was assessed using QUADAS-2 (Figure 2). Kundu et al. (2022) [6] and Luo et al. (2017) [4] raised concerns about spectrum bias in patient selection. Applicability concerns were noted for Genty et al. [5] (cardiothoracic ICU only) and Zhang et al. (exclusion of COPD and surgical patients) [7]. The two Song studies demonstrated the lowest overall risk of bias [2,3].

### 3.4. Diaphragm Thickening Fraction (DTF)

Five studies evaluated DTF as a predictor of liberation outcomes (Table 2). Reported cutoff values ranged from 26% to 50%, reflecting heterogeneity in patient populations and measurement techniques.

Song et al. (2022) [2] demonstrated that DTF < 30.09% predicted weaning failure with an AUC of 0.868 (95% CI 0.792–0.944), sensitivity 78.4%, and specificity 84.9% in 110 ICU patients. Genty et al. (2022) [5] evaluated DTFmax in a cardiothoracic ICU population using a derivation and validation cohort design; DTFmax ≥ 50% during T-piece SBT predicted weaning failure with an AUC of 0.94 in the derivation cohort and an AUC of 0.99 in the validation cohort. Notably, the direction of this cutoff—high DTFmax predicting failure—reflects excessive diaphragmatic work rather than weakness in this post-surgical population. Kundu et al. (2022) [6] reported an AUC of 0.64 for DTF < 26%, with high sensitivity (90.9%) but low specificity (37.0%), likely reflecting spectrum bias from post-SBT-only enrollment. Song et al. (2024) [3] identified DTF < 31% as an independent predictor of weaning failure (adjusted OR 23.96; *p* = 0.024). Zhang et al. (2024) [7] reported an AUC of 0.888 with a sensitivity of 91.2% and a specificity of 70.4%.

**Figure 3:**
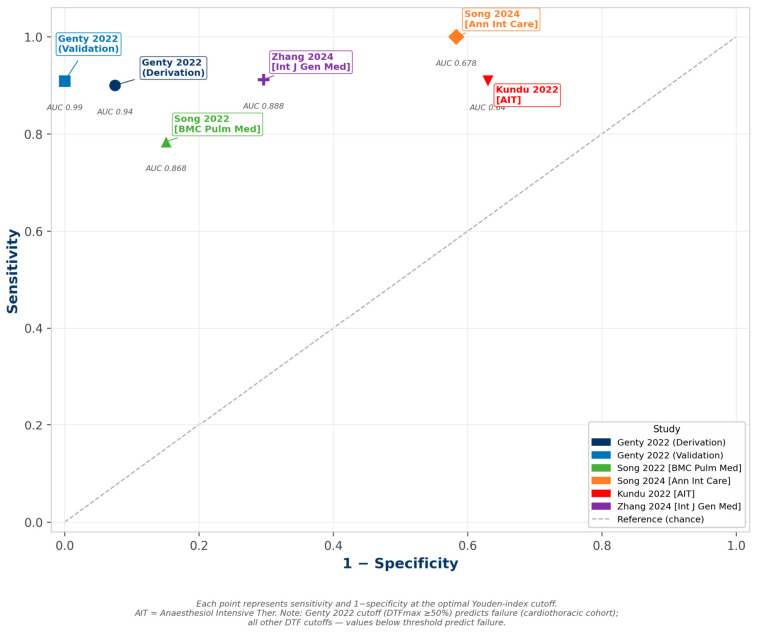
Diaphragm thickening fraction (DTF) ROC-style plot. Study-level sensitivity vs. 1−specificity at optimal Youden-index cutoff. Note: Genty 2022 cutoff (DTFmax ≥ 50%) predicts failure; all other DTF cutoffs—values below threshold predict failure. AIT = Anaesthesiol Intensive Ther. Data from [2,3,5,6,7].

Across the five studies, DTF demonstrated consistently high sensitivity (78–100%), though specificity varied substantially (37–100%) and the optimal diagnostic cutoff threshold remains unstandardized across populations and measurement protocols (Figure 3).

### 3.5. Diaphragmatic Excursion (DE)

**Figure 4:**
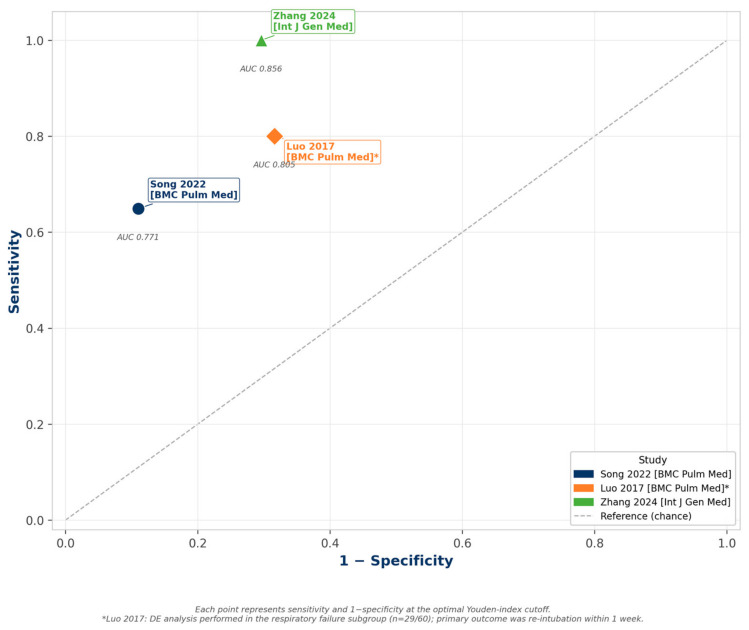
Diaphragmatic excursion (DE) ROC-style plot. Study-level sensitivity vs. 1−specificity at optimal Youden-index cutoff. * Luo 2017: DE analysis in the respiratory failure subgroup (*n* = 29/60); primary outcome was re-intubation within 1 week. Data from [2,4,7].

Three studies reported DE data. Song et al. (2022) [2] found DE <13.5 mm predicted weaning failure with an AUC of 0.771 (sensitivity 64.9%, specificity 89.0%). Luo et al. (2017) [4] demonstrated that a DE ≤ 12.6 mm predicted re-intubation within one week among a respiratory failure subgroup (AUC 0.805; sensitivity 80.0%, specificity 68.4%). Zhang et al. (2024) [7] reported an AUC of 0.856 with 100% sensitivity at DE >1.06 cm. Diagnostic thresholds and patient populations varied across studies, and results should be interpreted accordingly (Figure 4).

### 3.6. Lung Ultrasound (LUS)

**Figure 5:**
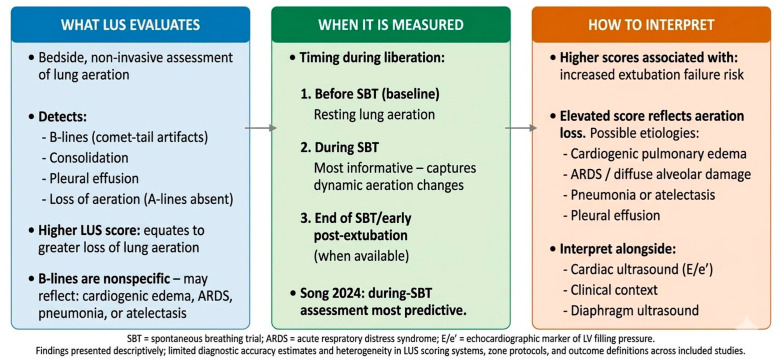
Descriptive summary of lung ultrasound (LUS) findings during spontaneous breathing trials. Higher LUS scores reflect aeration loss from multiple possible etiologies including ARDS, pneumonia, atelectasis, and cardiogenic edema. Data from [3,6]. SBT = spontaneous breathing trial; ARDS = acute respiratory distress syndrome.

Two studies evaluated LUS scores during SBTs. Kundu et al. (2022) [6] demonstrated that higher LUS scores were associated with extubation failure, reflecting loss of lung aeration. Elevated LUS scores are nonspecific—they may reflect cardiogenic pulmonary edema, ARDS-related diffuse alveolar damage, pneumonia, atelectasis, or pulmonary fibrosis—and the underlying etiology cannot be determined from the LUS score alone [3,6]. Song et al. (2024) [3] identified an antero-lateral LUS score > 7 as an independent predictor of weaning failure (AUC 0.721; sensitivity 73.3%, specificity 75.0%) and found no correlation between LUS scores and echocardiographic markers of LV filling pressure (E/e’). LUS results should be interpreted in conjunction with clinical assessment and, where available, cardiac ultrasound findings (Figure 5).

### 3.7. Summary of Findings and Diagnostic Accuracy

Table 2 presents structured diagnostic accuracy data from all included studies. DTF demonstrated the most consistent and highest discriminative performance (AUC range 0.64–0.99), followed by DE (AUC 0.77–0.86) and LUS (AUC 0.72–0.79).

## 4. Discussion

This systematic review synthesizes available evidence evaluating diaphragm and lung ultrasound as adjunctive tools in the assessment of liberation from mechanical ventilation. Across the six included studies, DTF showed the most consistent association with liberation failure, while LUS provided complementary information regarding pulmonary aeration. These findings are hypothesis-generating and should be interpreted in the context of a small, heterogeneous evidence base derived entirely from observational studies with modest sample sizes.

### 4.1. Physiologic Interpretation

Prolonged mechanical ventilation can lead to ventilator-induced diaphragmatic dysfunction characterized by muscle atrophy, reduced contractility, and impaired neuromuscular coupling. DTF provides a non-invasive surrogate of diaphragmatic contractile activity, while DE reflects global diaphragm displacement influenced by multiple factors beyond muscle strength alone. Lung ultrasound complements diaphragm assessment by identifying reduced lung aeration [3,6]. It is critical to recognize, however, that liberation failure is a complex, multifactorial process. In addition to respiratory muscle dysfunction and impaired lung aeration, failure may be driven by cardiac dysfunction, weaning-induced pulmonary edema, neurological impairment including delirium and central nervous system depression, renal dysfunction, metabolic derangements, and other systemic factors. Diaphragm and lung ultrasound address only a subset of these mechanisms—primarily respiratory muscle performance and lung aeration—and do not provide a comprehensive evaluation of all contributors to liberation failure. Conclusions regarding clinical applicability must therefore account for this inherent limitation.

### 4.2. Comparison with Conventional Weaning Indices

RSBI and NIF are established, widely validated, and clinically practical tools that remain integral to standard liberation assessment. Their limitations are well recognized but should not be overstated: they continue to perform adequately in the majority of extubation decisions across diverse ICU settings. The present review does not support the conclusion that diaphragm or lung ultrasound should replace or supersede these conventional indices. Rather, several included studies suggested that ultrasound-derived parameters may provide incremental information in selected patients, particularly when conventional indices alone leave clinical uncertainty [2,3,6]. It is important to note that ultrasound is itself operator-dependent, time-intensive, and not universally available, which limits its applicability as a routine first-line tool. Novel integrative indices such as DTF-RSBI (respiratory rate/DTF) and DE-RSBI (respiratory rate/DE) combine ultrasound with conventional parameters rather than replacing them. Song et al. (2022) reported a DTF-RSBI AUC of 0.859 vs. a conventional RSBI AUC of 0.639 in a single-center cohort [2]; Zhang et al. (2024) confirmed a DTF-RSBI AUC of 0.858 in a separate cohort [7]. These findings are preliminary and require prospective validation before clinical adoption.

### 4.3. Threshold Heterogeneity, Timing, and the Influence of ICU Subtype and SBT Method

A key observation from Table 2 is the substantial variation in optimal DTF diagnostic thresholds across studies, ranging from <26% (Kundu 2022 [6]) to ≥50% (Genty 2022 [5], predicting failure by excessive diaphragmatic work). Several factors likely contribute to this heterogeneity and warrant explicit consideration. First, ICU subtype materially influences the physiologic context: Genty et al. [5] enrolled an exclusively cardiothoracic surgical population in whom post-operative phrenic nerve injury, sternotomy pain, and pleural effusions are prevalent—conditions that alter the expected relationship between DTF and liberation success in ways that do not apply to general medical ICU populations. This likely explains both the reversed cutoff direction and the unusually high AUC (0.94–0.99) in that study. Second, the SBT method affects the contribution of ventilator-driven flow to diaphragm movement. During pressure support ventilation (PSV), DE and DTF reflect a combination of active diaphragmatic contraction and passive lung inflation driven by the ventilator; during T-piece or minimal support SBTs, measurements more purely reflect patient effort. This distinction is particularly relevant for DE, and may account for the lower and more variable specificity observed in PSV-based trials. Third, the timing of ultrasound assessment—before, during, or at the end of the SBT—captures different physiologic states. Measurements taken during SBT may reflect dynamic diaphragmatic fatigue developing over the course of the trial, whereas pre-SBT measurements capture resting function. Song et al. (2024) found that measurements taken during SBT were most informative [3], consistent with the hypothesis that dynamic assessment captures relevant changes not apparent at rest. These factors collectively indicate that DTF thresholds are not generalizable across populations and settings, and that any future clinical protocol will need to be validated within the specific ICU context and SBT method in which it is intended to be used.

### 4.4. Proposed Framework and Future Research Agenda

**Figure 6:**
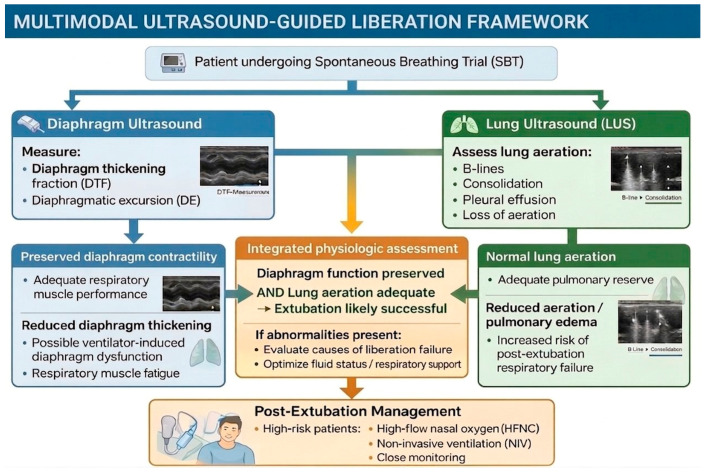
Proposed hypothesis-generating framework for multimodal ultrasound assessment during spontaneous breathing trials. This conceptual model is intended to illustrate how diaphragm and lung ultrasound findings could be integrated alongside conventional indices in future prospective evaluations. It does not represent a validated or evidence-based clinical protocol [2,3,4,5,6,7]. DTF = diaphragm thickening fraction; DE = diaphragmatic excursion; LUS = lung ultrasound; HFNC = high-flow nasal cannula; NIV = non-invasive ventilation.

The framework presented in Figure 6 is proposed as a hypothesis-generating conceptual model, not as a clinically validated or evidence-based protocol. Based on the current evidence—six small observational studies with heterogeneous protocols—it would be premature to advocate for the integration of diaphragm and lung ultrasound into routine clinical liberation algorithms. The available data are insufficient to support recommendations regarding specific ultrasound thresholds, the timing of assessment, or the superiority of ultrasound-guided over conventional liberation approaches. Future prospective interventional studies are needed to determine whether ultrasound-guided liberation assessment improves patient-centered outcomes such as extubation failure rates, duration of mechanical ventilation, or ICU length of stay. Multicenter randomized trials with standardized protocols and predefined ultrasound thresholds would be required to move this conceptual framework toward clinical applicability.

### 4.5. Limitations

This review has several important limitations that must be considered when interpreting the findings. First, only six studies comprising 430 patients in total were included, with individual study sample sizes ranging from 51 to 120 patients. These sample sizes are modest, and the observed diagnostic accuracy estimates—particularly AUC values—carry wide confidence intervals and are likely subject to optimism bias in single-center observational designs. Second, all included studies are observational; none evaluated ultrasound-guided liberation as an intervention compared with standard care. Observational diagnostic accuracy data cannot establish whether incorporating ultrasound into clinical decision-making would actually improve outcomes. Third, there was substantial heterogeneity in ultrasound acquisition protocols, diagnostic thresholds, SBT methods, and outcome definitions across studies, precluding pooled meta-analysis and limiting cross-study comparisons; a formal diagnostic test accuracy meta-analysis with sROC curves was not conducted for this reason and remains a priority for future work. Fourth, publication bias was not formally assessed (e.g., using Deeks’ funnel plot asymmetry test) due to the small number of included studies (fewer than ten), which renders such tests unreliable; however, the possibility that negative or null studies remain unpublished cannot be excluded. Fifth, a formal GRADE assessment of the certainty of evidence was not conducted, as GRADE-DTA frameworks require pooled estimates that could not be robustly derived given the heterogeneity across studies; the certainty of evidence should therefore be considered low to very low pending larger standardized studies. Sixth, ultrasound is inherently operator-dependent: image quality and measurement reproducibility are sensitive to operator training and experience, and between-operator variability was not systematically reported in any included study. Seventh, liberation failure is a multifactorial outcome influenced by respiratory, cardiac, neurological, renal, and metabolic factors; diaphragm and lung ultrasound address only a subset of these mechanisms. Eighth, two studies had specific QUADAS-2 concerns: Kundu et al. enrolled only post-SBT-success patients (spectrum bias), and Luo et al. [4] reported DE only in a respiratory failure subgroup. Genty et al. [5] conducted the study exclusively in a cardiothoracic ICU, limiting generalizability to mixed medical-surgical populations. Finally, the review protocol was not prospectively registered, which limits methodological transparency. These limitations collectively indicate that the current evidence base is preliminary and insufficient to support definitive clinical recommendations.

### 4.6. Future Directions

Several high-priority research needs emerge from this review. First, there is currently no universally adopted protocol for diaphragm ultrasound assessment during liberation from mechanical ventilation. The development and prospective validation of a standardized acquisition and measurement protocol—specifying probe type, anatomical landmark, respiratory phase timing, and number of measurements to average—is a prerequisite for meaningful cross-study comparison, meta-analysis, and eventual clinical guideline development. Second, as the literature matures and standardized protocols emerge, a formal diagnostic test accuracy meta-analysis with pooled sensitivity and specificity estimates and summary ROC (sROC) curves would substantially strengthen the evidence base beyond what descriptive synthesis alone can provide. Third, prospective interventional studies are needed to determine whether ultrasound-guided liberation assessment improves patient-centered outcomes such as extubation failure rates, duration of mechanical ventilation, and ICU length of stay compared with conventional assessment. Multicenter randomized trials with prospectively registered protocols and predefined ultrasound thresholds would be required to move the conceptual framework proposed in this review toward clinical applicability. From a clinical implications standpoint, the present findings suggest that diaphragm and lung ultrasound may be most valuable as adjunctive bedside tools in patients whose conventional indices leave clinical uncertainty—such as those with prolonged mechanical ventilation, high-risk comorbidities (cardiac dysfunction, COPD, or neuromuscular disease), or recurrent extubation failure—rather than as universal screening tools for all ICU patients undergoing SBTs. Key challenges to clinical implementation include the absence of a validated standard protocol, operator dependency requiring dedicated training, limited availability of ultrasound expertise in lower-resource settings, and the need to integrate ultrasound findings meaningfully alongside conventional indices and clinical judgment. Addressing these challenges through training programs, protocol standardization efforts, and implementation science research will be as important as generating additional diagnostic accuracy data.

## 5. Conclusions

This systematic review evaluated the available evidence on diaphragm and lung ultrasound as adjunctive tools in the assessment of liberation from mechanical ventilation. DTF showed the most consistent association with liberation failure across included studies, while DE and LUS provided additional but less consistent information. These findings should be interpreted with caution: the evidence base consists of six small, heterogeneous observational studies, and no study demonstrated that ultrasound-guided assessment improves clinical outcomes compared with conventional indices. RSBI, NIF, and standard clinical assessment remain the established foundation of liberation practice. Diaphragm and lung ultrasound may have potential as complementary adjunctive tools in selected patients or research settings, but their role in routine clinical decision-making is not yet established. The proposed framework is hypothesis-generating and intended to inform future research priorities rather than guide immediate clinical practice. Standardization of measurement protocols, prospective validation in diverse populations, and ultimately randomized trials evaluating ultrasound-guided liberation protocols will be necessary before firm clinical recommendations can be made.

## Figures and Tables

**Table 1:** Characteristics of included studies.

Study (Year)	Design	Population (*n*)	ICU Type	SBT Method	US Modality	Timing	Outcome Definition	Follow-Up
Song 2022 [2]	Prospective cohort	110	Mixed	PSV 8/PEEP 0, 30 min	DTF, DE	End SBT	SBT failure or NIV/MV ≤ 48 h	≤48 h
Song 2024 [3]	Prospective cohort	51	Mixed	PSV 8, 120 min	DTF, LUS	During SBT	Reintubation or NIV ≤ 48 h	≤48 h
Luo 2017 [4]	Retrospective cohort	60 *	Mixed	T-piece, 30–45 min	DE	Post-SBT	Respiratory failure/reintubation	≤48 h/7 d
Genty 2022 [5]	Prospective (deriv.+valid.)	89	CT-ICU	T-piece, 30–60 min	DTFmax	During SBT	SBT failure or reintubation ≤ 48 h	≤48 h
Kundu 2022 [6]	Prospective cohort	60 †	Mixed	PSV 5/PEEP 5, 120 min	DTF, LUS	Pre- and post-SBT	NIV or invasive MV ≤ 48 h	≤48 h
Zhang 2024 [7]	Prospective observational	120	Mixed	PSV-based, 30–120 min	DTF, DE	During SBT	Reintubation or NIV after extubation	≤48 h

DTF: diaphragm thickening fraction; DE: diaphragmatic excursion; LUS: lung ultrasound; PSV: pressure support ventilation; SBT: spontaneous breathing trial; NIV: non-invasive ventilation; CT-ICU: cardiothoracic ICU. * Luo 2017 [4]: DE analysis in respiratory failure subgroup (*n* = 29/60). † Kundu 2022: enrolled only post-SBT-success patients [6].

**Table 2:** Diagnostic accuracy of diaphragm and lung ultrasound parameters for predicting liberation failure.

Study (Year)	Parameter	*n* (Fail/Total)	Cut-Off	AUC (95% CI)	Sensitivity (%)	Specificity (%)	PPV (%)	NPV (%)
DIAPHRAGM THICKENING FRACTION (DTF)								
Song 2022 [BMC Pulm Med] [2]	DTF	37/110	<30.09%	0.868 (0.792–0.944)	78.4	84.9	72.5	88.6
Genty 2022 Derivation [Respir Care] [5]	DTFmax	10/50	≥50%	0.94 (0.89–0.99)	90.0	92.5	NR	NR
Genty 2022 Validation [Respir Care] [5]	DTFmax	11/39	≥50%	0.99 (0.97–1.00)	90.9	100	NR	NR
Kundu 2022 [Anaesthesiol Intensive Ther] [6]	DTF	27/60	<26%	0.64 (NR)	90.9	37.0	NR	NR
Song 2024 [Ann Intensive Care] [3]	DTF	15/51	<31%	0.678 (0.532–0.823)	100	41.7	41.7	100
Zhang 2024 [Int J Gen Med] [7]	DTF	27/95	>31.11%	0.888 (0.807–0.943)	91.2	70.4	NR	NR
DIAPHRAGMATIC EXCURSION (DE)								
Song 2022 [BMC Pulm Med] [2]	DE	37/110	<13.5 mm	0.771 (0.664–0.877)	64.9	89.0	74.9	83.3
Luo 2017 [BMC Pulm Med] [4] *	DE (avg)	10/29	≤12.6 mm	0.805 (NR)	80.0	68.4	NR	NR
Zhang 2024 [Int J Gen Med] [7]	DE	27/95	>1.06 cm	0.856 (0.769–0.919)	100	70.4	NR	NR
LUNG ULTRASOUND (LUS)								
Kundu 2022 [Anaesthesiol Intensive Ther] [6]	LUS score change	27/60	ΔLUS ≥ 1	0.79 (composite)	NR	NR	NR	NR
Song 2024 [Ann Intensive Care] [3]	Antero-lateral LUS	15/51	>7	0.721 (0.545–0.897)	73.3	75.0	55.0	87.1
Song 2024 [Ann Intensive Care] [3]	Global LUS score	15/51	>14	0.719 (0.564–0.875)	86.7	61.1	48.1	91.7

Δ = change from baseline; AUC = area under ROC curve; CI = confidence interval; DE = diaphragmatic excursion; DTF = diaphragm thickening fraction; DTFmax = higher DTF of two hemidiaphragms; LUS = lung ultrasound; NR = not reported; PPV/NPV = positive/negative predictive value. * Luo 2017 [4] reflects respiratory failure subgroup (n = 29); primary outcome was re-intubation within 1 week.

## Data Availability

Extracted data are available from the corresponding author upon reasonable request.

## Supplementary Materials

The following supporting information can be downloaded at: https://www.mdpi.com/article/10.3390/jcm15103877/s1. Supplementary Material S1: Search strategy (MEDLINE, EMBASE, Cochrane CENTRAL). Supplementary Material S2: PRISMA-DTA statement. Supplementary Material S3: QUADAS-2 risk of bias assessment methodology.
