# Supplementary material for: Bedside Diaphragm and Lung Ultrasound for Predicting Liberation from Mechanical Ventilation: A Systematic Review with Clinical Synthesis
Source: J Clin Med. 2026 May 18;15(10):3877. doi: 10.3390/jcm15103877 (PMC13207019; doi:10.3390/jcm15103877)
Supplement: Supplementary file 1 [file jcm-15-03877-s001.zip › jcm-4198916-supplementary.pdf]

Supplementary Materials

Supplementary Material 1. Search strategy

Databases searched: MEDLINE (PubMed), EMBASE, Cochrane CENTRAL

Final search date: January 20, 2026

MEDLINE (PubMed)

("mechanical ventilation"[MeSH Terms] OR ventilat*[Title/Abstract] OR intubat*[Title/Abstract]) AND (wean*[Title/Abstract] OR extubat*[Title/Abstract] OR "spontaneous breathing trial"[Title/Abstract] OR SBT[Title/Abstract]) AND (diaphragm[Title/Abstract] OR diaphragmatic[Title/Abstract]) AND (ultrasound[Title/Abstract] OR ultrasonograph*[Title/Abstract] OR sonograph*[Title/Abstract])

Filters: Humans; Adults (≥18 years)

EMBASE

('mechanical ventilation'/exp OR ventilat*:ti,ab OR intubat*:ti,ab) AND (wean*:ti,ab OR extubat*:ti,ab OR 'spontaneous breathing trial':ti,ab OR sbt:ti,ab) AND (diaphragm:ti,ab OR diaphragmatic:ti,ab) AND (ultrasound:ti,ab OR ultrasonograph*:ti,ab OR sonograph*:ti,ab)

Cochrane CENTRAL

(mechanical ventilation OR ventilat* OR intubat*) AND (wean* OR extubat* OR "spontaneous breathing trial" OR SBT) AND (diaphragm OR diaphragmatic) AND (ultrasound OR ultrasonograph* OR sonograph*)

Supplementary Material 2. PRISMA-DTA statement

This systematic review and diagnostic test accuracy meta-analysis was conducted and reported in accordance with the PRISMA-DTA statement.

Supplementary Material 3. QUADAS-2 risk of bias assessment

Risk of bias was assessed independently by two reviewers using the QUADAS-2 tool across the following domains: patient selection, index test, reference standard, and flow and timing. Overall risk of bias across included studies was low to moderate. Applicability concerns were primarily related to heterogeneity in ultrasound acquisition protocols, timing during spontaneous breathing trials, and outcome definitions across studies.
